# Exploiting Smart Meter Power Consumption Measurements for Human Activity Recognition (HAR) with a Motif-Detection-Based Non-Intrusive Load Monitoring (NILM) Approach

**DOI:** 10.3390/s21238036

**Published:** 2021-12-01

**Authors:** Sebastian Wilhelm, Jakob Kasbauer

**Affiliations:** Deggendorf Institute of Technology, 94469 Deggendorf, Germany; jakob.kasbauer@th-deg.de

**Keywords:** non-intrusive load monitoring (NILM), human activity recognition (HAR), smart meter, ambient assisted living (AAL), motif search, ambient intelligence (AmI), internet of things (IoT)

## Abstract

Numerous approaches exist for disaggregating power consumption data, referred to as non-intrusive load monitoring (NILM). Whereas NILM is primarily used for energy monitoring, we intend to disaggregate a household’s power consumption to detect human activity in the residence. Therefore, this paper presents a novel approach for NILM, which uses pattern recognition on the raw power waveform of the smart meter measurements to recognize individual household appliance actions. The presented NILM approach is capable of (near) real-time appliance action detection in a streaming setting, using edge computing. It is unique in our approach that we quantify the disaggregating uncertainty using continuous pattern correlation instead of binary device activity states. Further, we outline using the disaggregated appliance activity data for human activity recognition (HAR). To evaluate our approach, we use a dataset collected from actual households. We show that the developed NILM approach works, and the disaggregation quality depends on the pattern selection and the appliance type. In summary, we demonstrate that it is possible to detect human activity within the residence using a motif-detection-based NILM approach applied to smart meter measurements.

## 1. Introduction

To provide residents with feedback about the power consumption of the appliances in their households, and to motivate them to save energy, numerous organizations and researchers are working on methods to monitor household power consumption, in detail, at the appliance level [1,2,3,4]. These methods are called Appliance Load Monitoring (ALM). Essentially, there are two different approaches to ALM. One is Intrusive Load Monitoring (ILM), which means that each appliance is monitored individually by sensing, for example, with the help of individual power plugs at each appliance. This method provides precise data, but the installation of such a system is complex and expensive [2,4]. The second approach is called Non-Intrusive Load Monitoring (NILM) or Non-Intrusive Appliance Load Monitoring (NIALM). In NILM, aggregated power consumption data, for example, of a whole residential building or several circuits, are considered and disaggregated using specific algorithms to infer the operation of individual appliances. NILM is more cost-effective and easier to deploy than ILM because it requires only a single sense point, but disaggregating the data is a current challenge. Due to many different appliances that can be present in a household, and the overlapping of different appliances in the total power consumption, it is still not possible to fully disaggregate the total power consumption of a household [1,2,3,4,5].

The objective of our work is also to disaggregate the aggregated power consumption of a household, but not for energy monitoring, which most of the related work addresses, but to be able to detect human activities in residence. With the help of the activity data of the residents, Ambient-Assisted Living (AAL) systems can be implemented later on, which can recognize untypical behaviors of the residents, which, for example, indicate a need for help, an emergency, or a decrease in the health status [6]. As a source for the aggregated data, we focus on measurements from commercial smart meters. The currently available smart meters can provide power consumption data with a resolution of up to 1 Hz [5,7]. Reusing the data of commercial smart meters as a data source has the advantage that no proprietary sensors have to be installed for Human Activity Recognition (HAR) [6].

Disaggregating power consumption data to use the appliance activity information in an AAL system results in special requirements, usually not considered in NILM literature. In contrast to projects with the objective of energy monitoring, it is particularly important to optimize precision and recall of appliance activity identification for disaggregating data for emergency detection in AAL systems. Otherwise, potential emergencies may be detected too late.

Furthermore, it is essential to focus on appliances with direct human interaction (e.g., coffee machines, TVs, hairdryers) and not on typical energy guzzlers that consume electricity without human intervention (e.g., freezers, heat pumps).

A further aspect we want to address is to develop a NILM method that allows the disaggregation on an edge system in (near) real-time, performed in the residential environment (e.g., on a single board computer). Thus, the power consumption data do not have to be sent to third parties, which improves privacy. Furthermore, there is no need for an (constantly) active internet connection, which has further advantages regarding the system’s availability.

The main contribution of this paper is a new approach for NILM, which uses pattern-recognition and motif-search on the raw power waveform to recognize individual household appliances on the total power consumption of a household, measured by the smart meter. In other words, to detect from the aggregated smart meter readings which specific appliances are active at a particular time. Our method is designed for (near) real-time appliance activity detection in a streaming setting using edge computing. We quantify the spotting uncertainty using continuous pattern correlation instead of using binary device activity states. The disaggregated appliance activity stream serves as the basis for HAR.

The remaining paper is structured as follows: we review the NILM literature in Section 2 and outline how NILM is already used in the HAR context. In Section 3, we introduce our new NILM approach based on pattern detection and evaluate this approach in Section 4 using the ‘*GeLaP*’ dataset [8]. We then outline the transformation of the disaggregated power consumption data to information on human activity based on the literature in Section 5 and conduct a practical case study. The paper ends with a discussion in Section 6, and a conclusion and outlook in Section 7.

## 2. Related Work

Investigations in the field of NILM can be traced back to George W. Hart, who presented a prototype for a *‘nonintrusive appliance load monitor’* in 1985 [9]. Hart’s prototype can track residential electricity consumption in a *‘nonintrusive and inexpensive manner’* by installing a microprocessor-based unit on a household’s electricity meter.

Since then, numerous researchers and organizations have been working on various approaches for NILM. By 22 August 2019, Liu [4] identified 617 relevant scientific documents in the field of NILM. Most of the work focuses on breaking down the energy consumption of individual household appliances and, thus, motivating the residents to save energy in order to reduce emissions [1,2,4,10].

Further approaches exist that use NILM as a Home Energy Management System (HEMS) to compensate peak loads and, thus, ensure grid stability (e.g., charging the batteries of electric cars primarily in off-peak times) [2,11], to detect appliance malfunction [1,11], to detect energy theft [1], or for HAR in the AAL domain [1,12,13,14,15,16].

Common NILM approaches usually operate in four steps: *data acquisition*, *event detection*, *feature extraction*, and *load identification*. Thereby the approaches significantly differ from each other in various dimensions. These are mainly: • the sample rate, collection method, and included features of the raw data [1,2,10,11,17]; • the implementation of event detection, where expert heuristics, probabilistic models, or matched filters, among others, are frequently used [2,11]; • the selected features for appliance identification [2,17]; • the methods of learning and inference (supervised, semi-supervised, and unsupervised) [2,10].

Several surveys provide a detailed overview of NILM: in particular, the frequently cited work of Zeifman & Roth [3] from 2011 and Zoha et al. [2] from 2012 are noteworthy. More recent, but less frequently cited, surveys in the NILM context are the works of Sadeghianpourhamami et al. from 2018 [17], Ruano et al. from 2019 [11], or Bonfigli & Squartini from 2020 [18].

- Zeifman & Roth provides a fundamental overview of NILM. The paper’s primary focus is the presentation of different methods for feature extraction, divided into low-frequency and high-frequency measurements. The authors claim that *‘no complete NIALM solution suitable for all types of household appliances is available’*. Further, they conclude that *“No complete set of robust, widely accepted appliance features has been identified"* [3].
- Zoha et al. present the principle of NILM, but at a more detailed level than the work of Zeifman & Roth mentioned previously. The authors present the four basic steps of NILM, focusing on appliance features (specifically steady-state and transient-state) as well as learning and inference in NILM systems by presenting both supervised and unsupervised methods. Overall, Zoha et al. draw the same conclusions as Zeifman & Roth. In addition, the authors remark that future research should focus on unsupervised methods, since labeling data, as required for supervised methods, is not practical [2].
- Sadeghianpourhamami et al. focus on appliance feature selection and provides a categorization of state-of-the-art features [17].
- Ruano et al. provide a comprehensive overview of NILM in the application fields of HEMS and AAL. The paper also includes a summary of the main characteristics of 25 different NILM approaches in terms of sample rate, features, and their methodologies for load identification [11].
- Bonfigli & Squartini provide a broad overview of the current state of the art regarding NILM, along with a review of publicly available datasets and used evaluation metrics. The book focuses on approaches based on Hidden Markov Model (HMM) or Deep Neural Network (DNN) since the authors consider these approaches the most promising due to their capability and performance [18].

Throughout reviewing the mentioned surveys [2,3,11,17,18], we noticed that, although there has been research in NILM for more than 35 years, there are still many open challenges. One of the essential issues is the selection of the predictive features. Features on the P−Q plane are most commonly used [17]. Thus, recognition of high-power ON–OFF devices works rather well, but low-power devices often cannot be identified through the circuit’s noise [2,17]. Existing approaches, suitable for recognizing multi-state-appliances or continuous-state appliances are mainly based on supervised learning methods. However, these approaches requires large labeled data sets and often high-frequency sample rate for the learning phase, making the approaches non-scalable [2,3,11,17]. Nevertheless, other research avoid collecting their own data set, and studies mainly refer to existing data sets, such as *REDD* [19] or *UK-DALE* [11,20]. However, for a method that can also be applied in practice, it is necessary to consider data collection, storage, and processing. Approaches using low-frequency smart meter data [21,22] or edge computing [23] are rare.

Further, privacy concerns of residents arise from the practical deployment of NILM, as disaggregated power consumption data can be used to create detailed activity profiles [2,11,24,25,26].

Within this work, we intend to use NILM for HAR in private households and use this information in future work to implement an AAL system for detecting emergencies. In recent years, several studies have already been carried out in this context. One of the first activities is the *AUTAGEF* project, in which a demonstrator was developed to detect unusually long periods of inactivity in residences based on aggregated power consumption data. However, this demonstrator has so far not been evaluated in a real-life environment [21,27,28].

The works of Alcalá et al. [13], and Chalmers et al. [29] show that it is possible to identify common person-specific behaviors and deviations from them at the disaggregated power consumption. For this purpose, Alcalá et al. conducted case studies on the *UK-DALE* and *HES* datasets. The authors excluded data disaggregation and instead refers to related work [13]. Chalmers et al. investigated whether smart meter measurements can be used to detect behavioral changes of individuals that might indicate dementia. Unfortunately, the paper does not clarify precisely how the appliance recognition is implemented. The authors conclude that it may be possible to detect dementia using NILM, but the evaluation conducted in two households is insufficient for a general statement [29].

Patrono et al. [14,15] present a hybrid approach for HAR using power consumption data by combining smart meter measurements for appliances with high-power consumption and smart plugs for low-power appliances. However, the practical evaluation of the approach is pending.

Pascher [30] and Bousbiat et al. [16] are concerned with the practical implementation of activity recognition systems based on smart meter measurements. Pascher focuses on the conception of a software architecture to transfer the power consumption data from the households to a central server system for data analysis and emergency detection [30]. From an implementation perspective, the work of Bousbiat et al. is particularly noteworthy. The authors are trying to implement activity detection via edge computing performed in the household. For this purpose, they retrieve the smart meter data directly from the household, store it in a local database, and use the Grafana integration of the home automation software OpenHAB for disaggregation. Based on the events recognized by Grafena, the authors want to infer activities. However, the developments have so far only been tested in a laboratory instead of an actual household. Concurrently active appliances, or noise are not taken into account [16].

Further, more recent papers in the context of using NILM for HAR are found in the paper by Develin & Barry [25], and Ishizu et al. [31]. In 2019, Develin & Barry published a work that aimed to detect Activities of Daily Living (ADLs) using NILM, but it was not aimed to develop an AAL system; instead, they focused on providing detailed feedback to residents about their power consumption. Therefore, the disaggregation was primarily conducted on appliances with a high load consumption (1–2 kW or larger) using multi-layer, feedforward neural networks [25]. A paper by Ishizu et al. from 2020 investigates whether HAR data can be identified from the power measurements of a house. The authors use a supervised approach and restrict themselves to three activities (sleep, cooking, and go-out) [31].

To summarize, although there has been research in the field of NILM for more than 35 years, there are still major research gaps in the disaggregation of power consumption data. In particular, data collection, which serves as the basis for NILM, requires greater consideration [11]. Moreover, developing solutions that take into account the privacy of the residents should be considered [2,11,24,25].

Using disaggregated power consumption data for HAR is a recent trend. So far, there are mainly conceptual or proof-of-concept works; real-world implementation and evaluation are still pending.

## 3. Methodology for NILM

The NILM problem can be formulated as follows: let c(t) be the aggregated total power consumption of a household measured at the time *t*. The total power consumption in a household at the time *t* is the sum of the active power consumption of all electrical appliances:

(1) $$c\left(t\right)=\sum_{a_{i}\in A}c_{a_{i}}\left(t\right)$$

where *A* is the set of all electrical appliances in the residence, $c_{a_{i}}\left(t\right)$ is the individual consumption of the appliance $a_{i}\in A$ at the time *t*.

To refine the equation, we add a noise term n(t), which describes the noise of the electrical signal at time *t*. With this noise term, we are able to model measurement errors:

(2) $$c\left(t\right)=n\left(t\right)+\sum_{a_{i}\in A}c_{a_{i}}\left(t\right)$$

The objective of NILM is to infer individual consumers $a_{i}\in A$ from the aggregated dataset c(t)—to recognize appliances [2,18,32].

As described in Section 2, there are numerous approaches that address this problem, which differ in various dimensions. In this work, we introduce a new approach suitable for processing aggregated data, measured by commercial smart meters in (near) real-time to detect typical interactions with electrical appliances that are related to human activity. To address privacy concerns, the presented approach performs disaggregation on an edge computer (e.g., directly in the household) so that power consumption data do not reach third parties. Furthermore, we want to ensure the transferability of the approach into a real-world environment by relying on a pattern-detection method that does not require label data.

To develop and evaluate our approach, we recorded the ’*GeLaP’* dataset. This dataset contains aggregated power consumption data from 20 private households in Germany with a resolution of 1 Hz. The aggregated data were collected using a commercially available smart meter. In addition, the individual power consumption of 10 selected appliances in each household, where the electrical power consumption indicates direct human interaction, was collected with a resolution of up to 7 Hz. For this purpose, single-device-power-meters (intermediate power sockets) connected directly to the appliance to be measured were used [8].

The data set is publicly available in anonymized form at https://mygit.th-deg.de/tcg/GeLaP (accessed on 26 November 2021).

It is a fact that the peak power consumption and the temporal pattern in the power consumption time series are characteristic features for a specific electrical appliance [21,33]. With this work we intend to exploit this fact and recognize appliances by detecting the individual power pattern in the total power consumption of a household. For this purpose, however, it is first necessary to extract relevant power patterns from the individual power measurements. We term the extracted patterns in the raw power waveform as motifs. The motifs are extracted independently of the actual recognition. Therefore, it is possible to identify the motifs of a specific appliance in any household, requiring only the smart meter once the motifs are determined.

After pre-processing the raw measurements in Section 3.1, we identify repeating appliance power patterns (motifs) via time series clustering, in Section 3.2. We search in the aggregated smart meter power stream for motif occurrences, as described in Section 3.3.

### 3.1. Label Data Pre-Processing

In the ’*GeLaP’* dataset, the single-device-power-meter recordings provide timestamps for the request ($timestamp_{requ}$) and reply ($timestamp_{repl}$) for each sample, which is caused by *HTTP*-based recording architecture [8]. We choose the mean of $timestamp_{requ}$ and $timestamp_{repl}$ as unified timestamp because the electrical measurement happened between $timestamp_{requ}$ and $timestamp_{repl}$.

Furthermore, we downsample the separate recordings with varying the time axis and sample rates to a common time axis at a 1 Hz sample rate.

Additionally, we compensate for a constant lag between the single device power meter recordings and the smart meter recordings. The compensation maximizes the correlation between the recordings of different meters, by varying the lag length. Aligning both time axes increases the label quality for our evaluation (see Section 4).

Moreover, we fill up recording gaps by linear interpolation if the gap is shorter than 5% of the motif length *w*. Interpolating small gaps reduces the number of sliding windows that contain NaN entries. A single NaN entry in a sliding window causes the pattern similarity computations to fail for the window. Interpolating short gaps keeps the overall pattern intact and even steep slopes are preserved. We do not interpolate long gaps.

Furthermore, we eliminate sections in the recordings with constant values by adding dithering noise to recordings. The noise magnitude is smaller than the quantization resolution of power. Therefore, the noise is too small to perturb the recordings. Sections with constant values would cause faulty similarity computations in motif spotting according to Section 3.3.

### 3.2. Motif Selection

There are multiple different repeating power patterns for each appliance $a_{i}\in A$. Due to the number of different appliances $a_{i}\in A$ and the number of different possible patterns per appliance, it is infeasible to manually annotate all power consumption patterns and events. Thus, we algorithmically identify the recurring patterns and events. Our approach is unsupervised *sliding window subsequence clustering* on the time series of power consumption of the single device power meters, which is provided by the *‘GeLaP’* dataset. The result is a grouping of distinctive and repeating temporal patterns (motifs) *M*, grouped by pattern similarity.

To quantify the similarity between subsequences within the power measurements of a specific device $c_{a_{i}}\left(t\right)$, we use the z-normalized euclidean distance and its dual, the Pearson correlation. The algorithms that compute these similarity measures are free of tunable parameters. Furthermore, the algorithms compute the measures exactly instead of approximating the measures. Whereas an approximated similarity measure could have caused false positives matches [34,35].

One pitfall of this similarity measure is the information loss of the power magnitude due to the z-normalization to 0-mean and 1-std. As a consequence, all step functions are normalized to the same shape. For example, the power waveform of activating a 60 W lamp or a 2 kW heater becomes normalized to the same waveform, the unit step function.

We compute the full distance matrix, which contains the pairwise distance for all possible pairwise comparisons of subsequences in $c_{a_{i}}\left(t\right),a_{i}\in A$. Afterwards, we transform the distance matrix into a weighted undirected graph. In the graph, the nodes are the timestamps of subsequences and the edge-weights are pairwise distances. The motif grouping emerges after we partition the graph via community detection [36].

**Figure 1:**
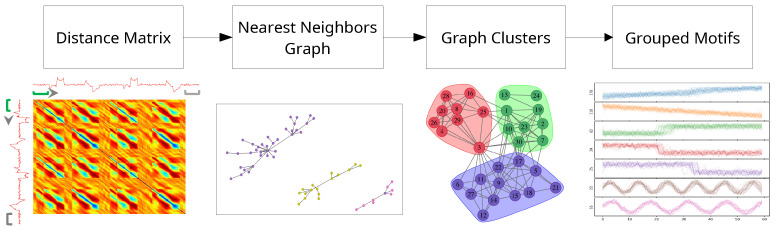
Flowchart of processing steps for unsupervised motif selection. First Image Source: Modified from Mueen & Eamonn [37]. Third Image Source: Modified from Ferreira & Zhao [36].

This process is outlined in Figure 1.

Due to the indistinguishability of singular step functions, we prefer cluster centers consisting of multiple steps or more complex waveforms. To quantify the oscillation information we use time series complexity [38].

We exclude timespans with a constant 0 power from the clustering to speed up the clustering.

For each appliance $a_{i}\in A$, the clustering extracts at least one motif $motif_{n}\in M$. We record the origin appliance for each motif in the form of a mapping $g:=motif_{n}\to a_{i}$, where $g\left(motif_{n}\right)=a_{i}$.

A parameter for the motif selection is the motif’s length. We set the motif length to 128 s (w=128), because 128 s is sufficiently long to capture the whole length of appliance operation modes. Additionally, 128 is a power of 2, which speeds up pairwise similarity computation.

### 3.3. Motif Spotting

The main objective of our work is to spot the clustered motifs $motif_{n}\in M$ in the aggregated smart meter measurements c(t), to determine when individual appliances $a_{i}\in A$ were active. For motif-spotting, we search the smart meter measurements for subsequences, which are similar to the motif sequences. For computing the similarity, we use Mueen’s ultra-fast Algorithm for Similarity Search (MASS), because MASS is algorithmically efficient and free of hyperparameters [39,40]. MASS computes the similarity between a query-sequence and all possible sliding window subsequences in a corpus-sequence. The output of MASS is a time series of similarities. We use the similarity as primary decision criteria for appliance activity detection.

In the following, we present the steps of our implementation of device recognition using the MASS algorithm based on the *‘GeLaP’* dataset.

#### 3.3.1. Smart Meter Measurements Pre-Processing

For motif detection using the MASS algorithm, it is required that the aggregated smart meter measurements are available in the same temporal resolution as the extracted motifs, which is 1 Hz in our case. It must also be ensured that the aggregated time series does not contain any gaps, otherwise the MASS algorithm cannot be applied successfully.

However, the aggregated data in the *‘GeLaP’* dataset are not consistently available in a resolution of 1Hz. Furthermore, there can be gaps in the smart meter measurements. Moreover, the smart meter time stamp increases by 1 ms after every approximately 20 samples, consequently the average resolution of the data is lower than 1 Hz [8].

We fill up gaps, similar to the label data pre-processing (Section 3.1) and compensate the shift in the time stamp by uniformly resampling the data, to bring the data to a resolution of exactly 1 Hz.

#### 3.3.2. Corresponding Line Conductor Detection

Within the *‘GeLaP’* dataset, the power consumption of each line conductor in the household was measured separately. The total household power consumption at time *t* is defined as:

(3) $$c\left(t\right)=\sum_{k=\{1,2,3\}}c_{lc_{k}}\left(t\right)$$

where $c_{lc_{k}}\left(t\right)$ is the active power consumption on line conductor *k* at the time *t*.

By monitoring the load profiles of each line conductor separately, instead of only the total power consumption c(t), the overlapping of several active appliances and noise can be reduced. Consequently, the untangling of c(t) facilitates the recognition [5].

Further, to save computing capacity and to exclude possible false positive detections on the other line conductors, we only intend to search for the appliance $a_{i}\in A$ on the line conductor k∈{1,2,3} to which the appliance $a_{i}$ is actually connected. Therefore, it is necessary to know to which line conductor a specific appliance is connected.

The recorded data in *‘GeLaP’* do not provide a mapping *f* from appliance $a_{i}$ to $lc_{k}$, so the mapping must therefore be inferred from recorded data. We use the following inference approach: An electrical appliance $a_{i}$, where $a_{i}\in A$ and *A* is the set of appliances in the residence, is always connected to at least one line conductor $lc_{k}$, where k∈{1,2,3}. Further, it is true, that an electrical appliance $a_{i}$ with a fixed connection is constantly connected to the same line conductor $lc_{k}$. For such appliances, the explicit correlation is valid: $f\left(a_{i}\right)=lc_{k}$. Consequently, a true positive match can only occur on this line conductor $lc_{k}$ during appliance recognition. It is trivial that if $f\left(a_{i}\right)=lc_{k}$, then the inverse also holds; that $f\left(a_{i}\right)\neq lc_{j}$, where j∈{1,2,3},j≠k.

Unless the motifs *M* are a disjoint set and an appliance $a_{i}$ is constantly connected to the same line conductor $lc_{k}$, a true positive match can only occur on that specific line conductor $lc_{k}$. We assume that for each appliance—provided that it was active at least once in the recording period—the maximum similarity occurs on the line conductor $lc_{k}$ (to which the appliance is actually connected to) and not on $lc_{j}$.

We were able to verify this observation with a few samples from households to which we also had physical access.

#### 3.3.3. Device Activity Spotting

We apply the MASS algorithm for each motif $motif_{n}\in M$ with the data of the corresponding line conductor $lc_{k}$: $c_{lc_{k}}\left(t\right)$. The result of MASS is a time series of similarities $SIM_{motif_{n}}\left(t\right)$ and indicates the similarity between $c_{lc_{k}}\left(t\right)$ at time *t* to the motif $motif_{n}$.

We infer that the appliance $a_{i}$ was active at time *t*, if the similarity $SIM_{motif_{n}}\left(t\right)$ is high.

**Figure 2:**
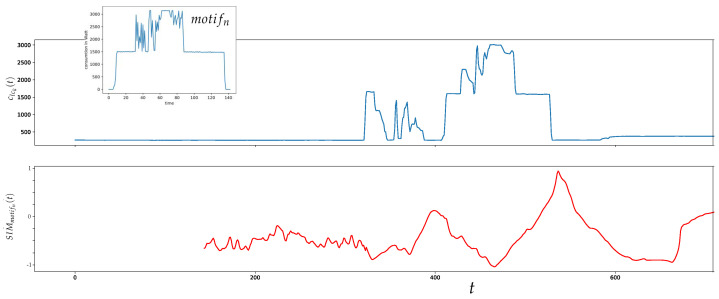
Example for a similarity time series $SIM_{motif_{n}}\left(t\right)$ generated by applying the MASS algorithm with an example motif $motif_{n}\in M$ on the corresponding line conductor $lc_{k}$ in a time period where the $motif_{n}$ was active once.

An example for a similarity time series generated by applying the MASS algorithm is outlined in Figure 2.

To reduce false positive matches, we employ a power heuristic: Because we know that all appliances $a_{i}\in A$ are power consumers and not generators, we infer that the line conductor power is the sum of *positive* power values. We model the line conductor power from time *t* to time t+w, where *w* is the window length as:

(4) $$c_{lc_{k}}\left(\left[t\;:\;\left(t\;\;+\;\;w\right)\right]\right)=\underset{\begin{array}{c} \mathrm{energy}\;\mathrm{of}\;\mathrm{noise}\\ \mathrm{of}\;\mathrm{the}\;\mathrm{electrical}\\ \mathrm{signal}\;\mathrm{from}\;\mathrm{time}\;\\ t\;\mathrm{to}\;\mathrm{time}\;t+w \end{array}}{\underset{︸}{n([t\;:\;(t\;\;+\;\;w)])}}+\underset{\begin{array}{c} \mathrm{total}\;\mathrm{energy}\\ \mathrm{of}\;\mathrm{the}\;\mathrm{motif}\;n \end{array}}{\underset{︸}{motif_{n}}}+\underset{\begin{array}{c} \mathrm{energy}\;\mathrm{of}\;\mathrm{all}\;\mathrm{other}\;\mathrm{appliances}\\ \mathrm{in}\;\mathrm{the}\;\mathrm{household}\;\mathrm{to}\;\mathrm{which}\;\mathrm{the}\\ \mathrm{motif}\;n\;\mathrm{does}\;\mathrm{not}\;\mathrm{correspond}\\ \mathrm{to}\;\mathrm{from}\;\mathrm{time}\;t\;\mathrm{to}\;\mathrm{time}\;t+w \end{array}}{\underset{︸}{\sum_{a_{h}\in \left\{A\setminus g\left(motif_{n}\right)\right\}}c_{a_{h}}\left(\left[t\;:\;\left(t\;\;+\;\;w\right)\right]\right)}}$$

Assuming that the background power $n\left(\left[t\;:\;\left(t\;\;+\;\;w\right)\right]\right)+\sum_{a_{h}\in \left\{A\setminus g\left(motif_{n}\right)\right\}}c_{a_{h}}\left(\left[t\;:\;\left(t\;\;+\;\;w\right)\right]\right)$ is slower changing than the motif pattern, we subtract the background power from the line conductor sliding window $c_{lc_{k}}\left(\left[t\;:\;\left(t\;\;+\;\;w\right)\right]\right)$. We compute the background power background(t) as a 3-h sliding window minimum.

In the case that several appliances (with similar power magnitudes) are concurrently active at timespan [t:(t+w)], the power waveform is perturbed and consequently, the pattern similarity will be low. In the other case, a high pattern similarity is not a necessary condition for an active appliance because the correlation is oblivious to the power magnitude, due to the z-normalization of MASS.

We construct the following Boolean time series as features for appliance detection:

(i) $max\left(motif_{n}\right)*tol\leq max\left(c_{lc_{k}}\left(\left[t\;:\;\left(t\;\;+\;\;w\right)\right]\right)\right)-background\left(t\right)$
  The maximum power consumption of a motif $motif_{n}$ (after deduction of a tolerance factor) must not be higher than the maximum power on the corresponding line conductor $lc_{k}$ in the period under consideration, from time *t* to time t+w subtracting the calculated background power for this period.
(ii) $min\left(motif_{n}\right)*tol\leq min\left(c_{lc_{k}}\left(\left[t\;:\;\left(t\;\;+\;\;w\right)\right]\right)\right)-background\left(t\right)$
  The minimum power consumption of a motif $motif_{n}$ (after deduction of a tolerance factor) must not be higher than the minimum power on the corresponding line conductor $lc_{k}$ in the period under consideration, from time *t* to time t+w, subtracting the calculated background power for this period.
(iii) $\left(max\left(motif_{n}\right)-min\left(motif_{n}\right)\right)*tol\leq max\left(c_{lc_{k}}\left(\left[t\;:\;\left(t\;\;+\;\;w\right)\right]\right)\right)-min\left(c_{lc_{k}}\left(\left[t\;:\;\left(t\;\;+\;\;w\right)\right]\right)\right)-background\left(t\right)$
  The span of the power of a motif $motif_{n}$ (after deduction of a tolerance factor) must not be higher than the span of the power on the corresponding line conductor $lc_{k}$ in the period under consideration, from time *t* to time t+w subtracting the calculated background power for this period.
(iv) $sum\left(motif_{n}\right)*tol\leq sum\left(c_{lc_{k}}\left(\left[t\;:\;\left(t\;\;+\;\;w\right)\right]\right)\right)-background\left(t\right)*w$
  The energy of a motif $motif_{n}$ (after deduction of a tolerance factor) must not be higher than the energy on the corresponding line conductor $lc_{k}$ in the period under consideration, from time *t* to time t+w, subtracting the background calculated power for this period.

where tol is a tolerance factor tol∈[0,1]. To give some slack, we set tol=0.9.

Each one of the above features is a necessary condition for appliance activity. Therefore we reject times by forcing the similarity to 0 at the times *t*, where one of these features is FALSE. This rejection of times reduces the number of false positive matches, caused by times with high similarity but without the necessary power consumption characteristics.

## 4. Evaluation of Motif-Detection-Based NILM

In Section 3, we introduced a new approach for NILM using motif detection. In the following, we evaluate our NILM approach based on the *‘GeLaP’* dataset.

The NILM literature distinguishes two primary types of errors that can occur by disaggregating power data [3,41,42]:

- Type I: false detection.
  The NILM algorithm detects that an appliance $a_{i}$ was active at time *t*, although it was actually not active. This is called false positive (FP) detection.
- Type II: missed detection.
  The NILM algorithm indicates that an appliance $a_{i}$ was not active at time *t*, although it was actually active. This is called false negative (FN) detection.

All correctly detected cases are referred to as true positive (TP); (it was correctly detected that an appliance $a_{i}$ was active at time *t*) or true negative (TN) (it was correctly indicated that an appliance $a_{i}$ was not active at time *t*).

This section first provides an overview of standard performance metrics used in NILM literature in Section 4.1. Since the regressor presented in this work differs from related approaches, specifically in the fact that the output of our regressor is not binary, but a time series of Pearson-R correlations, the common standard cannot be directly applied. Therefore, in Section 4.2, we present our approach to evaluating our NILM approach by exploiting the inter-observer reliability. Finally, we present and interpret the evaluation results in Section 4.3.

### 4.1. Evaluation Metric

In the NILM context, numerous performance metrics exist to evaluate the disaggregation approaches. An overview of standard metrics is provided by Pereira & Nunes [42].

Frequently, authors refer to calculating a confusion matrix and to determine the accuracy for a NILM algorithm [2,43]. However, Zoha et al. [2] and Makonin & Popowich [43] note that even the definition of accuracy is not consistent in the NILM context. Liang et al. [41] specify this issue and present in their work various accuracy measures, namely the *detection accuracy* ($\eta_{det}$), the *disaggregation accuracy* ($\eta_{dis}$), and the *overall accuracy* ($\eta_{all}$). In addition, Liang et al. propose considering the accuracy at the appliance level [41].

Another commonly used method for evaluating NILM approaches is the receive operating characteristics—area under curve (ROC–AUC) [41,42]. The ROC curve represents the dependency between true positive-rate (TPR) and false positive-rate (FPR), where TPR is defined as: $TPR=\frac{\#TP}{\#TP+\#FN}$ and FPR is defined as $FPR=\frac{\#FP}{\#FP+\#TN}$.

Because the individual appliances $a_{i}\in A$ are rarely active, there is an imbalanced ratio between positive and negative classes; therefore, this is referred to as an imbalanced classification problem. Consequently, the accuracy or the ROC–AUC is not a suitable metric for performance evaluation since even a classifier/regressor that states an appliance is off at any time would achieve a high accuracy or high ROC–AUC values [2,44,45]. The fact that accuracy or ROC–AUC is nevertheless often used as a performance metric is a limitation in many related papers.

Overall, it appears that there is no standardized approach for the performance evaluation of NILM [46].

To avoid this limitation, we use precision $P=\frac{\#TP}{\#TP+\#FP}$ and recall $R=\frac{\#TP}{\#TP+\#FN}$ as performance metrics. Precision and recall are not affected by the imbalanced class ratios.

Since our NILM approach differs from existing approaches by the facts that the output is not binary, and we further consider different motifs $motif_{n}\in M$ of an appliance $a_{i}$ independent from each other, it is not possible to directly determine #TP, #TN, #FN, and #FP. Therefore, it is necessary to introduce a specific methodology to calculate the confusion matrix, which is a basis to determine *P* and *R*. We present a complementary approach in the following Section 4.2.

### 4.2. Inter-Observer Reliability-Based Evaluation Metric

The *‘GeLaP’* dataset contains, for selected appliances $A^{\prime }\subseteq A$, individual power consumption measurements $c_{a_{i}}\left(t\right)$, $a_{i}\in A^{\prime }$ in addition to aggregated power measurements from each line conductor of the smart meter $c_{lc_{k}}\left(t\right)$, k∈{1,2,3} [8]. The individual measured values are collected independently of the aggregated data, but synchronized in time. If an arbitrary power-consuming event *e* occurs on any appliance $a_{j}\in A^{\prime }$, this is independently observed by both the individual power meter $c_{a_{j}}\left(t\right)$, connected directly to the appliance $a_{j}$ and the smart meter $c_{lc_{k}}\left(t\right)$, where $f\left(a_{i}\right)=lc_{k}$.

We exploit this independence of the observers for the evaluation, since with a perfectly working classifier/regressor, any event *e* at time *t* on an appliance $a_{j}\in A^{\prime }$, would have to be detected similarly in the individual power measurements $c_{a_{j}}\left(t\right)$ as well as from the aggregated measurements $c_{lc_{k}}\left(t\right)$, as by definition:

(5) $$c_{lc_{k}}\left(t\right)=c_{a_{j}}\left(t\right)+\sum_{a_{l}\in \left\{\tilde{A}\setminus a_{i}\right\}}\left(c_{a_{l}}\left(t\right)\right)+n\left(t\right)$$

where $\tilde{A}\subseteq A$ defines all appliances, connected to the line conductor $lc_{k}$.

Because we cannot detect arbitrary events *e* using our presented NILM approach, but rather spot for specific motifs $motif_{n}\in M$, we first determine our ground truth for the evaluation by applying the previously specified *devices activity spotting* approach on the down sampled (1 Hz) measurements of the individual power meters. Applying the algorithm for each motif $motif_{n}\in M$, with the respective individual power meter measurements belonging to the motif $motif_{n}$ results in a time series of Pearson-R correlations, which we interpret as ground truth $L_{motif_{n}}\left(t\right)$.

Since there exist some gaps in the individual power meter measurements $c_{a_{i}}\left(t\right)$, we ignore all time intervals with any gaps for the evaluation.

Having obtained the ground truth, we are now able to compare this ground truth $L_{motif_{n}}\left(t\right)$ individually with the output of the regressor on the aggregated measurements $SM_{motif_{n}}\left(t\right)$, for each motif $motif_{n}\in M$. It is required to consider each time *t* separately, thereby the following applies by definition:

(i) if $SM_{motif_{n}}\left(t\right)=L_{motif_{n}}\left(t\right)$ classify as true positive / true negative.
(ii) if $SM_{motif_{n}}\left(t\right)<L_{motif_{n}}\left(t\right)$ classify as false negative.
(iii) if $SM_{motif_{n}}\left(t\right)>L_{motif_{n}}\left(t\right)$ classify as false positive.

**Figure 3:**
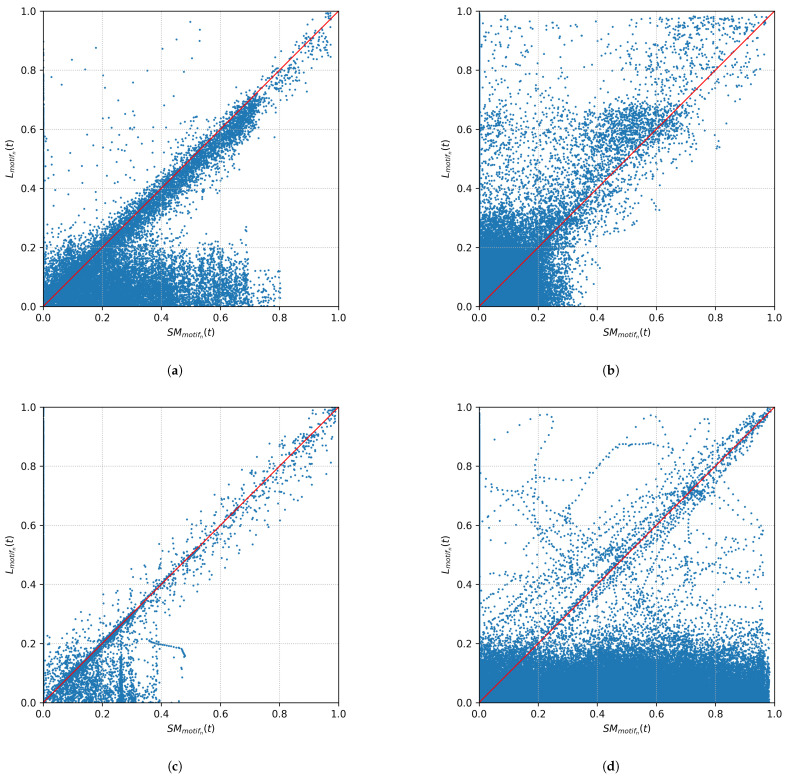
Scatter plot of correlations between $L_{motif_{n}}\left(t\right)$ and $SM_{motif_{n}}\left(t\right)$ for four exemplary motifs. (**a**) Motif of a fully automatic coffee machine (’GeLaP’: household 05; device: 003). (**b**) Motif of a washing machine (’GeLaP’: household 05; device: 007). (**c**) Motif of a water pump (’GeLaP’: household 05; device: 008). (**d**) Motif of a television (’GeLaP’: household 05; device: 009).

Figure 3 shows four exemplary plots for the point-by-point comparison of the regressor on $L_{motif_{n}}\left(t\right)$ and $SM_{motif_{n}}\left(t\right)$. Each point in the plot represents a specific time *t*. The red line represents a target line for correct classifications (TP/TN).

It is obvious from Figure 3 that the classification described above is too strict, since an exact match $SM_{motif_{n}}\left(t\right)=L_{motif_{n}}\left(t\right)$ is very uncommon, since due (measurement) noise already $SM_{motif_{n}}\left(t\right)\neq L_{motif_{n}}\left(t\right)$ can be observed.

There are two further properties that have to be considered within the evaluation; let $motif_{n}\in M$:

(i) for two times $t_{1}$ and $t_{2}$, where $t_{1}\neq t_{2}$, the absolute error at time $t_{1}$ is more valued than at time $t_{2}$, if:
  $$|SM_{motif_{n}}\left(t_{1}\right)-L_{motif_{n}}\left(t_{1}\right)|>|SM_{motif_{n}}\left(t_{2}\right)-L_{motif_{n}}\left(t_{2}\right)|$$
(ii) for two times $t_{1}^{\prime }$ and $t_{2}^{\prime }$, where $t_{1}^{\prime }\neq t_{2}^{\prime }$, the classification at time $t_{1}^{\prime }$ is to be valued higher than at time $t_{2}^{\prime }$, if:
  $$SM_{motif_{n}}\left(t_{1}^{\prime }\right)>SM_{motif_{n}}\left(t_{2}^{\prime }\right)$$

To involve these causalities in the evaluation and to obtain a less strict regression to determine true positive/true negative cases, we consider the following four sections, depending on thres∈[0;1]:

(i) $SM_{motif_{n}}\left(t\right)\geq thres$  and  $L_{motif_{n}}\left(t\right)\geq thres$  classify as true positive.
(ii) $SM_{motif_{n}}\left(t\right)<thres$  and  $L_{motif_{n}}\left(t\right)<thres$  classify as true negative.
(iii) $SM_{motif_{n}}\left(t\right)<thres$  and  $L_{motif_{n}}\left(t\right)\geq thres$  classify as false negative.
(iv) $SM_{motif_{n}}\left(t\right)\geq thres$  and  $L_{motif_{n}}\left(t\right)<thres$  classify as false positive.

**Figure 4:**
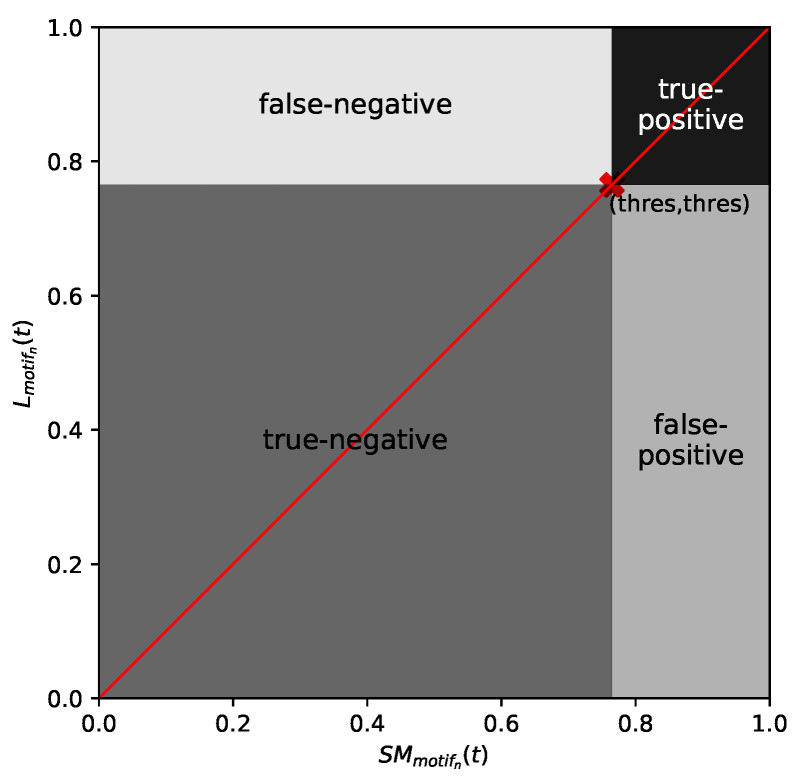
Exemplary division of the point-by-point comparison of the regressor on $L_{motif_{n}}\left(t\right)$ and $SM_{motif_{n}}\left(t\right)$ to a confusion matrix (thres=0.76).

These sections are sketched in Figure 4.

For our evaluation approach, however, we do not fix the value thres, but sweep in the interval from 0 to 1 over it (step: 0.001) and calculate *P* and *R* for each thres separately.

**Figure 5:**
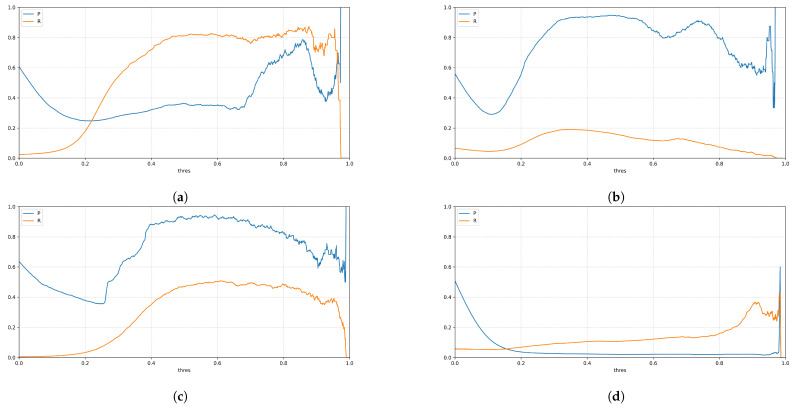
Precision $P_{motif_{n}}\left(thres\right)$ and Recall $R_{motif_{n}}\left(thres\right)$ for four exemplary motifs. (**a**) Motif of a *fully automatic coffee machine* (’GeLaP’: household 05; device: 003). (**b**) Motif of a *washing machine* (’GeLaP’: household 05; device: 007). (**c**) Motif of a *water pump* (’GeLaP’: household 05; device: 008). (**d**) Motif of a *television* (’GeLaP’: household 05; device: 009).

As an example, the swept *P* and *R* values for the motifs from Figure 3 are shown in Figure 5.

It is worth mentioning the behavior at the edge thres→1, here it can regularly be observed that $P_{motif_{n}}\to \infty$ and $R_{motif_{n}}\to 0$ applies. This behavior is due to the fact that the observer very rarely identifies $SM_{motif_{n}}\left(t\right)\to 1$ or $L_{motif_{n}}\left(t\right)\to 1$ and, therefore, there are only few positive cases for calculating *P* and *R*. This fluctuation at thres→1, is shown in Figure 5 where the precision spikes to 1 and recall dips to 0.

Overall, Figure 5 shows that the fully automatic coffee machine motif, for example, is detected more completely, i.e., consistently with higher recall than all other sample motifs. However, the fully automatic coffee machine achieves lesser precision than the motifs of the washing machine and the water pump.

For the television motif, it can be observed that both precision and recall indicate that the recognition does not work properly, which is in line with the intuitive assumption from Figure 3.

To compare the evaluation of the motifs amongst each other, we define the score $PS_{motif_{n}}^{a}$ as the median of all $P_{motif_{n}}\left(thres\right)$ values, where thres∈[a;1]. Analogously, we define $RS_{motif_{n}}^{a}$ as the median of all $R_{motif_{n}}\left(thres\right)$ values, where thres∈[a;1].

For the following analyses, we set a=0.5. $PS_{motif_{n}}^{a}$ and $RS_{motif_{n}}^{a}$ for the previous outlined example motifs are shown in Table 1.

### 4.3. Evaluation Results

For each appliance $a_{i}\in A^{\prime }$ from the *’GeLaP’* dataset (in summary, around 200 appliances), we repeat the evaluation method as described in Section 4.2 and calculated $PS_{motif_{n}}^{0.5}$ and $RS_{motif_{n}}^{0.5}$, $\forall motif_{n}\in M$.

**Figure 6:**
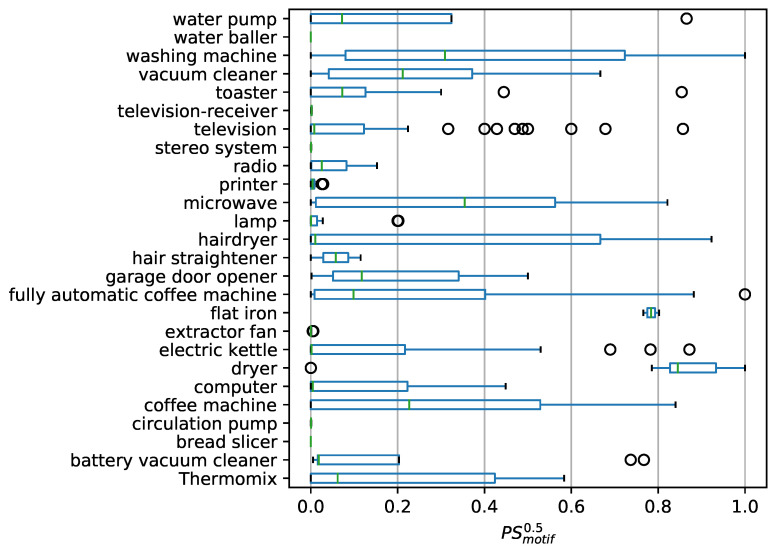
Box plot on $PS_{motif_{n}}^{0.5}$ for all extracted motifs $motif_{n}\in M$ grouped by appliance.

**Figure 7:**
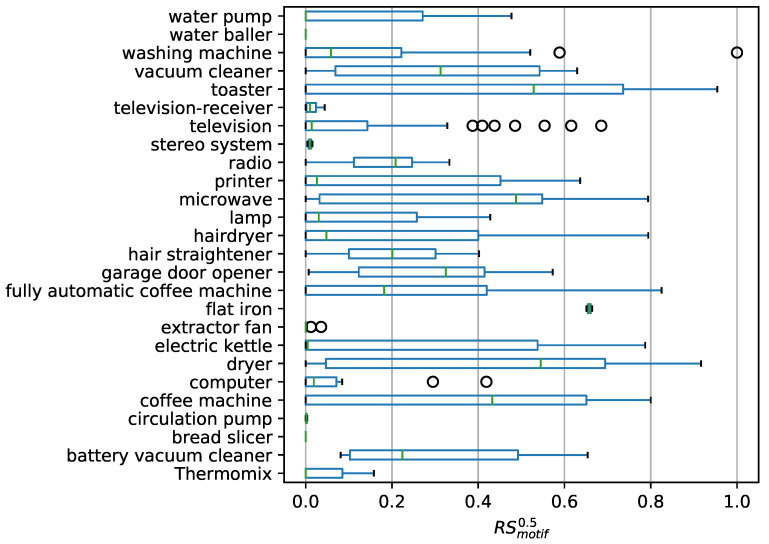
Box plot on $RS_{motif_{n}}^{0.5}$ for all extracted motifs $motif_{n}\in M$ grouped by appliance.

We group the appliances $a_{i}$ by appliance type and present $PS_{motif}^{0.5}$ and $RS_{motif}^{0.5}$ for each appliance group in a box plot in Figure 6 and Figure 7. Each appliance group consists of several physical appliances $a_{i}\in A^{\prime }$ across different households. Additionally, each physical appliances $a_{i}$ consists of several motifs $motif_{n}$, stemming from the unsupervised clustering (see Section 3.2).

Figure 6 and Figure 7 show that both precision and recall are highly dependent on the appliance group. We notice that some appliance groups are generally (almost) not detected ($PS_{AG_{i}}^{0.5}\to 0$) these are specifically: water baller, television receiver, stereo system, extractor fan, circulation pump, or bread slicer. For other appliance groups, the recall varies significantly even within the appliance group and there are motifs that are recognized with a high recall and others with a low recall. A particular example is the washing machine, where the minimum recall is 0 and the median is less than 0.1, but there is an outlier with $PS_{motif}^{0.5}=1$.

We observe an analogous situation for the precision; here, again, we consider the washing machine, for example, and observe that the plot spans the entire range between 0 and 1, with a median of 0.3.

This effect, both for recall and for precision, can be explained by the fact that within the appliance groups there are some motifs that can be spotted very well (high precision and/or high recall), and other motifs are recognized very weakly or not at all.

## 5. Detecting Human Actions Using NILM

Our work’s higher objective is to use the disaggregated power consumption data to detect human actions within the residence. After introducing a NILM approach in Section 3 and evaluating it in Section 4, we finally want to describe the transition to HAR in this section. Section 5.1 describes the theoretical approach for exploiting the disaggregated power consumption data for HAR before we conduct a specific case study with Section 5.2 based on the *‘GeLaP’* dataset.

First of all: in the context of HAR, the two terms ‘action’ and ‘activity’ are commonly used, which we would like to introduce briefly in this section, following Chen et al. [47]. An ‘action’ describes a simple, usually short-time, behavior executed by a single person (e.g., opening a door). On the other hand, an ‘activity’ refers to complex behaviors consisting of a sequence of actions and/or interleaving or overlapping actions (e.g., making a meal) [47]. Sensors only detect the ‘actions’ of the residents. However, there are already numerous methods in the literature for inferring from ‘actions’ to ‘activities’ like HMM, Hidden Semi Markov Model (HMM), or conditional random field (CRF) [48,49,50]. Within this work, we focus on detecting simple ‘actions’. Further processing of the data to identify specific ‘activities’ can be carried out in future work if required.

### 5.1. Theoretical Approach

The theoretical background for the recognition of human actions is provided by the works of Perkowith et al. [51], Philipose et al. [52], and Wyatt et al. [53]. The authors validated that it is possible to derive a human action from the interaction with specific objects/devices (e.g., the coffee machine). For validation, they tagged different objects with Radio Frequency Identification (RFID) tags and attached RFID readers on the wrists of the residents.

However, when transferring this knowledge to the disaggregated power consumption data, we have to consider that there are different appliances, which partly also change their states, even without a human interacting with them. We identify the following types of appliances:

Type I Fully manual appliances:
  appliances that change their operation state only after user interaction, such as simple on-off operations.
Type II Partial automated appliances:
  appliances that react to user interaction, but also partially perform autonomous functions (e.g., automatic switch-off, cleaning program).
Type III Fully Automated appliances/stand alone appliances:
  fully automated appliances that change state without any user interaction (e.g., in the smart home) or appliances that are continuously active and self-regulating to change state (e.g., refrigerator).

For unambiguously inferring human actions from the disaggregated power consumption data, only those state changes of the individual appliances shall be interpreted as human actions initiated by direct human interaction with the corresponding appliance. For Type I appliances, any state change—in our case, this means any motif detection—can be immediately interpreted as human action.

For Type II appliances, it is necessary to consider the individual events, i.e., the individual motifs, in a differentiated manner. Only those state changes exclusively triggered by human interaction with the appliance shall be interpreted as human actions without any restrictions. Status changes that are performed (partially) autonomously, i.e., without human intervention (e.g., sleep timer/energy saving shutdown), may not be considered unlimited as activity signals. Depending on the use case, it is possible to completely ignore such status changes as signals for human action or interpret them further using device-specific logic operations. However, it is necessary to obtain more detailed information about the motifs for Type II appliances. For each motif, it must be determined whether the underlying appliance activity is generated unambiguously by human interaction with the appliance or whether a (partially) autonomous appliance function can also generate the specific power consumption pattern.

Type III appliances are not suitable for detecting human actions.

### 5.2. Case Study

To illustrate how our approach works, i.e., in detecting human actions in residences, based on disaggregated power consumption data, we conducted a case study in the following matter. For this purpose, we refer, as an example, to the data from the *‘GeLaP’* dataset for household 07 in the time period from 28 October to 3 November 2019.

According to the metadata provided by Wilhelm et al. [8], the residence is located in Bavaria (Germany), has a size of >150 m${}^{3}$, and ≤2 persons live in it. *‘GeLaP’* contains submetered data from a total of ten appliances in the residence, which are described in Table 2.

In the context of this case study, we will only consider the first three appliances $\bar{A}$ (namely: fully automatic coffee machine, microwave, and electric kettle), as these are characterized by a particularly high load intensity, and are, therefore (with the knowledge from Section 4) considered to be particularly suitable for disaggregation.

According to Section 3.2, the first step is extracting common motifs $\bar{M}$ from the submetered data of appliances $\bar{A}$.

After the extraction of the common motifs $\bar{M}$, it has to be determined manually for Type II devices by adding external knowledge, which, of the extracted motifs, can be unambiguously assigned to a human activity $\bar{M^{\prime }}\subseteq \bar{M}$. In our case, this step has to be carried out exclusively for the fully automatic coffee machine. Expert knowledge made it possible to identify all motifs that could be caused by an automatic process (e.g., switch-off process). These motifs are then excluded in $\bar{M^{\prime }}$.

**Figure 8:**
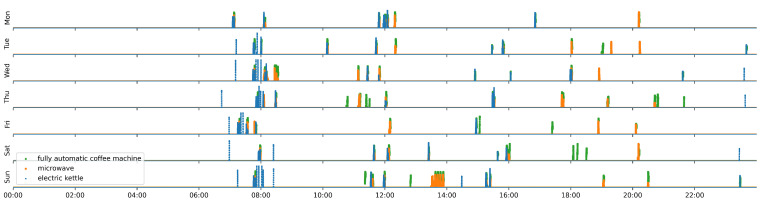
Human activity profile of household 07 of the *’GeLaP’* dataset in the period from 28 October to 3 November 2019, which was created using the presented NILM methodology on the smart meter data for the appliances fully automatic coffee machine, microwave, and electric kettle. Values between 0 and 1 are possible on the Y-axis, which show the Pearson-R correlation of the corresponding motifs to the aggregated data. The higher the value, the better the agreement between the motif and the aggregated power.

Finally, it is now possible to detect the selected motifs $\bar{M^{\prime }}$ directly from the smart meter measurements $c_{lc_{k}}\left(t\right)$, k∈{1,2,3} by using the sporting approach we presented in Section 3.3. The detection results for 7 days are summarized in Figure 8.

Figure 8 shows directly that no human activities can be identified between approximately 00:00 a.m. and 06:30 a.m. during the observed period for interacting with the appliances *fully automatic coffee machine*, *microwave*, and *electric kettle*. During the morning hours (between approximately 06:30 a.m. and 08:30 a.m.), regularly performed human activities can be detected and during lunchtime (between 11:00 a.m. and 12:30 p.m.). In the afternoon and evening, activities of the resident are also present, but much more irregularly.

Overall, we already obtain a comprehensive activity profile of the household based on disaggregated data from the smart meter based on three appliances.

Further case studies are available in the  Supplementary Material and Section 7.

## 6. Discussion

In contrast to most common NILM approaches, which work event-based [2,10,11,54], we developed a *non-event-based* approach. The common processing steps event detection, feature extraction, and load identification are not executed separately, but in one processing step. It is continuously checked in the series of the total power measurements $c_{lc_{k}}\left(t\right)$, k∈{1,2,3} whether a known motif $motif_{n}\in M$ may have been spotted and, thus, an appliance activity—respectively, a human action—can be detected.

In general, non-event-based approaches are not as computationally efficient as event-based approaches, but they do not rely on edge detection schemes before classification. This, in turn, they have advantages, in that the type of identifier does not have to be determined in advance. Thus, this method can represent a more generic approach to NILM than an event-based NILM method.

The application of our approach requires continuous spotting for all known motifs *M* to determine whether any motif $motif_{n}\in M$ could be present at time *t*. Although the MASS comparison algorithm runs in O(nlogn) [39], this can cause performance problems as the number of appliances to be recognized, i.e., the number of possible motifs, increases. One way to prevent this problem would be to determine which appliances $\hat{A}$ are potentially available in a specific household and then just spot only for motifs $\hat{M}$, which corresponds to appliances $a_{j}\in \hat{A}$. Assuming that the number of electrical appliances in a household is limited and that only a subset of the existing appliances is relevant for NILM, the calculation effort can be significantly reduced.

A key aspect in the design of our NILM approach was to be able to perform the disaggregation of smart meter measurements on an edge system directly in the residential environment (e.g., on a single board computer) in (near) real-time. Once the motifs have been identified (see Section 3.2), performed independently of the household, but only in relation to the appliance, the spotting methodology presented in Section 3.3 can be applied to an edge system. For spotting, it is only necessary to cache the aggregated data for as long as the corresponding motifs are long—so it does not require supervised systems or large databases of historical data. In concrete terms, this means that it is possible to cache the aggregated data directly in the household (e.g., using a ring buffer) and perform motif spotting in a streaming setting. Thus, human activity is detected directly in the household, and it is no longer necessary to transfer the aggregated data to a remote server. For multi-state appliances, in particular, this approach extends to the current state-of-the-art, as described in Section 2.

However, since our approach is based on motif spotting, full real-time activity detection is not achievable. In order to perform spotting of a specific $motif_{n}\in M$ of motif-length *w* by analyzing the most recent time window, it is always necessary that *w* new samples of the aggregated data are in the cache. Thus, when an appliance activity occurs, there is a delay of up to *w* seconds until the power consumption pattern arrives in the ring-buffer. Due to the constant lag, we refer strictly to (near) real time in this article.

Using an edge system has two major advantages: On the one hand, the system is not dependent on a (stable) internet connection. On the other hand, privacy and security concerns can be overcome, as neither consumption data nor activity data must be transmitted to third parties, which is considered an important aspect of the literature [2,11,24,25].

To interpret the performance evaluation for our NILM approach from Section 4.3, we created a stratified random guessing baseline observer by permuting on the time axis of $SM_{motif}\left(t\right)$. This results in a baseline performance for random guessing, which is approximately 0.01 for precision and recall, because the appliances are inactive 99% of the time. Consequently, we can state that our NILM approach works better than random guessing for a number of appliances.

However, it can also be noted that some appliance types can be better recognized than other types. This can be explained by the fact that appliances, such as the *fully automatic coffee machine*, which achieves high precision and recall, produce more distinctive patterns in their power consumption than constant low-power appliances, such as a *stereo system*. Variations in the quality of recognition (concerning precision and recall) can also be observed within the same appliance group. Even within the same physical appliance, there are performance variations across the motifs. One reason explaining this quality fluctuation is that the unsupervised clustering (see Section 3.2) does not incorporate knowledge about the appliance operating mode. Consequently, the clustering does not pre-select the motifs.

For assessing the evaluation, it is also worth mentioning that, across the threshold sweep, we use the median performance instead of the maximum performance. The precision and recall could be higher if we would choose the optimal threshold value. However, the performance curve across the threshold sweep is spiky and fluctuates at thresh→1. Furthermore, the optimal value for precision and recall occur at different threshold values. However, the advantage of our median evaluation methodology is that this is fully deterministic and has no dependency on cross-validation. Thus, the performance of our NILM approach is evaluated in a more generalized way.

The main focus of our work is to use the disaggregated NILM information to recognize human activity within the residence. The information about the human activity will later be used to develop AAL systems that can identify atypical behaviors of the residents that indicate, for example, a need for assistance, an emergency, or a decrease in health [6]. We were able to achieve this objective, as the case studies in Section 5.2 show. However, it was necessary to manually assign the motifs to human activities (especially for Type II appliances) through expert knowledge, as there are no ground truth data for human activities in the *‘GeLaP’* dataset.

Contrary to Patrono et al. [14,15] or Pascher [30], our approach differentiates essentially in the fact that we do not perform NILM or HAR on a central server or a cloud system. However, our approach can be applied directly in the household on an edge system. Our work follows the conceptual paper of Bousbiat et al. [16] instead. However, in contrast to Bousbiat et al., we do not use simulated data for our analysis, but rather data from actual households using the *’GeLaP’* dataset. Thus, our work is more practice-oriented with concerning HAR.

It is noteworthy that our NILM algorithm has no binary outputs. Therefore we cannot binarize HAR, but rather give weighted assumptions about the presence or absence of human activity. This “non-binary” differentiates our research from any related work.

## 7. Conclusions and Further Work

This paper presented a novel approach for NILM, which uses the pattern recognition/motif search on the raw power waveform to recognize individual household appliances on the total power consumption of a household measured by the smart meter. The approach is based on extracting repeating patterns—so-called motifs—of specific household appliances from individual power measurements of the appliance and spotting the motifs in the aggregated power consumption measurement of the household. This spotting is done using similarity search with the MASS algorithm and finally results in continuous Pearson-R correlations, which quantify the uncertainty of the disaggregation. Our approach enables disaggregation on an edge system in (near) real-time in a streaming setting, directly in the residential environment, which means that power consumption data do not have to be sent out of the house for processing.

However, the evaluation of the developed NILM approach shows that the quality of the disaggregation (in terms of precision and recall) strongly depends on the motif extraction and the selection of the relevant motifs spotted.

The further objective of the work was to use the disaggregated power consumption data for HAR. Based on the literature, we were able to show that the information on device activity can be transferred to human activity information using simple semantics. In this context, we conducted a case study in Section 5.2 with selected motifs for one exemplary household from the *‘GeLaP’* dataset.

In summary, we demonstrated that it is possible to detect human activity within the household using a motif-detection-based NILM approach applied to smart meter data.

In further work, the motif extraction should be revised and/or a preprocessing should be introduced to exclude those motifs that are recognized only insufficiently (low precision or low recall) and, thus, increase the overall quality of the NILM approach. Furthermore, a global database of ’known motifs’ should be built up so that our NILM approach can be transferred easily, since the complex pre-processing/motif extraction process is eliminated.

Subsequently, our NILM approach should be verified with other datasets and benchmarked against common (event-based) NILM methods. Furthermore, in a further step, the presented NILM approach should be integrated into a real-world environment and applied cumulatively using ring-buffered smart meter data.

With the help of advanced algorithms, the inferred data on human activity can be used for intelligent AAL systems, e.g., for emergency detection, which does not require proprietary sensors for HAR.

## Figures and Tables

**Table 1:** $PS_{motif_{n}}^{a}$ and $RS_{motif_{n}}^{a}$ for four exemplary motifs.

Appliance	${\mathrm{PS}}_{{\mathrm{motif}}_{n}}^{a}$	${\mathrm{RS}}_{{\mathrm{motif}}_{n}}^{a}$
fully automatic coffee machine	0.44	0.81
washing machine	0.83	0.1
water pump	0.87	0.48
television	0.02	0.14

**Table 2:** Submetered appliances in the household 07 including the assignment of device type.

Appliance	Type
Fully automatic coffee machine	Type II
Microwave	Type I
Electric kettle	Type I
Vacuum cleaner	Type I
Floor lamp	Type I
Computer	Type II
Printer	Type II
Television	Type II
Floor lamp	Type I
Radio	Type I

## Data Availability

The ‘*GeLaP*’ dataset, used in the evaluation, is described in Wilhelm et al. [8]. Available online at https://mygit.th-deg.de/tcg/gelap (accessed on 26 November 2021).

## Supplementary Materials

Further case studies, like Figure 8, are available online as supplementary material at https://mygit.th-deg.de/tcg/HAR-NILM (accessed on 26 November 2021).

## Abbreviations

The following abbreviations are commonly used in this manuscript:

AAL	ambient-assisted living
NILM	non-intrusive load monitoring
HAR	human activity recognition
ADL	activity of daily living
MASS	Mueen’s ultra-fast algorithm for similarity search

## References

[1] Herrero J.R., Murciego Á.L., Barriuso A.L., de la Iglesia D.H., González G.V., Rodríguez J.M.C., Carreira R. (2017). Non Intrusive Load Monitoring (NILM): A State of the Art. Advances in Intelligent Systems and Computing.

[2] Zoha A., Gluhak A., Imran M., Rajasegarar S. (2012). Non-Intrusive Load Monitoring Approaches for Disaggregated Energy Sensing: A Survey. Sensors.

[3] Zeifman M., Roth K. (2011). Nonintrusive appliance load monitoring: Review and outlook. IEEE Trans. Consum. Electron..

[4] Liu H. (2020). Non-Intrusive Load Monitoring.

[5] Marchiori A., Hakkarinen D., Han Q., Earle L. (2011). Circuit-Level Load Monitoring for Household Energy Management. IEEE Pervasive Comput..

[6] Wilhelm S. (2021). Exploiting Home Infrastructure Data for the Good: Emergency Detection by Reusing Existing Data Sources. Advances in Intelligent Systems and Computing.

[7] Wilhelm S. Activity-monitoring in Private Households for Emergency Detection: A Survey of Common Methods and Existing Disaggregable Data Sources. Proceedings of the 14th International Joint Conference on Biomedical Engineering Systems and Technologies.

[8] Wilhelm S., Jakob D., Kasbauer J., Ahrens D. GeLaP: German Labeled Dataset for Power Consumption. Proceedings of the Sixth International Congress on Information and Communication Technology-ICICT 2021.

[9] Hart G.W. (1985). Prototype Nonintrusive Appliance load Monitor: Progress Report 2.

[10] Zhuang M., Shahidehpour M., Li Z. An Overview of Non-Intrusive Load Monitoring: Approaches, Business Applications, and Challenges. Proceedings of the 2018 International Conference on Power System Technology (POWERCON).

[11] Ruano A., Hernandez A., Ureña J., Ruano M., Garcia J. (2019). NILM Techniques for Intelligent Home Energy Management and Ambient Assisted Living: A Review. Energies.

[12] Cardinaux F., Brownsell S., Hawley M., Bradley D. (2008). Modelling of Behavioural Patterns for Abnormality Detection in the Context of Lifestyle Reassurance. Lecture Notes in Computer Science.

[13] Alcalá J., Ureña J., Hernández Á., Gualda D. (2017). Assessing Human Activity in Elderly People Using Non-Intrusive Load Monitoring. Sensors.

[14] Patrono L., Primiceri P., Rametta P., Sergi I., Visconti P. An innovative approach for monitoring elderly behavior by detecting home appliance’s usage. Proceedings of the 2017 25th International Conference on Software, Telecommunications and Computer Networks (SoftCOM).

[15] Patrono L., Rametta P., Meis J. Unobtrusive detection of home appliance’s usage for elderly monitoring. Proceedings of the 2018 3rd International Conference on Smart and Sustainable Technologies (SpliTech).

[16] Bousbiat H., Klemenjak C., Leitner G., Elmenreich W. Augmenting an Assisted Living Lab with Non-Intrusive Load Monitoring. Proceedings of the 2020 IEEE International Instrumentation and Measurement Technology Conference (I2MTC).

[17] Sadeghianpourhamami N., Ruyssinck J., Deschrijver D., Dhaene T., Develder C. (2017). Comprehensive feature selection for appliance classification in NILM. Energ. Build..

[18] Bonfigli R., Squartini S. (2020). Machine Learning Approaches to Non-Intrusive Load Monitoring.

[19] Kolter J.Z., Johnson M.J. (2011). REDD: A public data set for energy disaggregation research. Workshop on Data Mining Applications in Sustainability (SIGKDD).

[20] Kelly J., Knottenbelt W. (2015). The UK-DALE dataset, domestic appliance-level electricity demand and whole-house demand from five UK homes. Sci. Data.

[21] Hildebrandt D., Schmidt F. AutAGef—Schlussbericht. https://slub.qucosa.de/api/qucosa%3A4170/attachment/ATT-0/.

[22] Buchmann E., Böhm K., Burghardt T., Kessler S. (2012). Re-identification of Smart Meter data. Pers. Ubiquit. Comput..

[23] Hernandez A., Nieto R., Fuentes D., Urena J. Design of a SoC Architecture for the Edge Computing of NILM Techniques. Proceedings of the 2020 XXXV Conference on Design of Circuits and Integrated Systems (DCIS).

[24] Greveler U. (2016). Die Smart-Metering-Debatte 2010–2016 und ihre Ergebnisse zum Schutz der Privatsphäre. Datenbank Spektrum.

[25] Devlin M.A., Hayes B.P. (2019). Non-Intrusive Load Monitoring and Classification of Activities of Daily Living Using Residential Smart Meter Data. IEEE Trans. Consum. Electron..

[26] Molina-Markham A., Shenoy P., Fu K., Cecchet E., Irwin D. Private memoirs of a smart meter. Proceedings of the 2nd ACM Workshop on Embedded Sensing Systems for Energy-Efficiency in Building.

[27] Clement J., Ploennigs J., Kabitzsch K. (2012). Smart Meter: Detect and Individualize ADLs. Ambient Assisted Living.

[28] Clement J., Ploennigs J., Kabitzsch K. (2013). Detecting Activities of Daily Living with Smart Meters. Ambient Assisted Living.

[29] Chalmers C., Fergus P., Curbelo Montanez C.A., Sikdar S., Ball F., Kendall B. (2020). Detecting Activities of Daily Living and Routine Behaviours in Dementia Patients Living Alone Using Smart Meter Load Disaggregation. IEEE Trans. Emerg. Top. Comput..

[30] Pascher M. (2020). Praxisbeispiel Digitalisierung konkret: Wenn der Stromzähler weiß, ob es Oma Gut Geht. Beschreibung des Minimalinvasiven Frühwarnsystems “ZELIA”. Wege in Die Digitale Zukunft: Was Bedeuten Smart Living, Big Data, Robotik & Co für Die Sozialwirtschaft?.

[31] Ishizu K., Mizumoto T., Yamaguchi H., Higashino T. Home Activity Recognition Using Aggregated Electricity Consumption Data. Proceedings of the 2020 IEEE International Conference on Smart Computing (SMARTCOMP).

[32] Hart G. (1992). Nonintrusive appliance load monitoring. Proc. IEEE.

[33] Müller K.J. (2010). Gewinnung von Verhaltensprofilen am intelligenten Stromzähler. DuD.

[34] Mueen A., Nath S., Liu J. (2010). Fast approximate correlation for massive time-series data. Proceedings of the 2010 ACM SIGMOD International Conference on Management of Data (SIGMOD ’10).

[35] Yeh C.C.M., Zhu Y., Ulanova L., Begum N., Ding Y., Dau H.A., Silva D.F., Mueen A., Keogh E., Bonchi F., Domingo-Ferrer J., Baeza-Yates R., Zhou Z., Wu X. (2016). Matrix Profile I: All Pairs Similarity Joins for Time Series: A Unifying View That Includes Motifs, Discords and Shapelets. Proceedings of the 2016 IEEE 16th International Conference on Data Mining (ICDM).

[36] Ferreira L.N., Zhao L. (2016). Time series clustering via community detection in networks. Inform. Sci..

[37] Mueen A., Keogh E. Time Series Data Mining Using the Matrix Profile: A Unifying View of Motif Discovery, Anomaly Detection, Segmentation, Classification, Clustering and Similarity Joins. Proceedings of the 23rd ACM SIGKDD Conference on Knowledge Discovery and Data Mining.

[38] Batista G.E., Wang X., Keogh E.J. A Complexity-Invariant Distance Measure for Time Series. Proceedings of the 2011 SIAM International Conference on Data Mining.

[39] Mueen A., Zhu Y., Yeh M., Kamgar K., Viswanathan K., Gupta C., Keogh E. (2017). The Fastest Similarity Search Algorithm for Time Series Subsequences under Euclidean Distance. http://www.cs.unm.edu/~mueen/FastestSimilaritySearch.html.

[40] Law S. (2019). STUMPY: A Powerful and Scalable Python Library for Time Series Data Mining. JOSS.

[41] Liang J., Ng S.K., Kendall G., Cheng J.W. (2010). Load Signature Study—Part I: Basic Concept, Structure, and Methodology. IEEE Trans. Power Deliv..

[42] Pereira L., Nunes N. (2018). Performance evaluation in non-intrusive load monitoring: Datasets, metrics, and tools—A review. Wires Data Min. Knowl. Discov..

[43] Makonin S., Popowich F. (2014). Nonintrusive load monitoring (NILM) performance evaluation. Energ. Effic..

[44] Kim H., Marwah M., Arlitt M., Lyon G., Han J. Unsupervised Disaggregation of Low Frequency Power Measurements. Proceedings of the 2011 SIAM International Conference on Data Mining. Society for Industrial and Applied Mathematics.

[45] Thai-Nghe N., Gantner Z., Schmidt-Thieme L. A new evaluation measure for learning from imbalanced data. Proceedings of the 2011 International Joint Conference on Neural Networks.

[46] Anderson K.D., Berges M.E., Ocneanu A., Benitez D., Moura J.M. Event detection for Non Intrusive load monitoring. Proceedings of the IECON 2012—38th Annual Conference on IEEE Industrial Electronics Society.

[47] Chen L., Hoey J., Nugent C., Cook D., Yu Z. (2012). Sensor-Based Activity Recognition. IEEE Trans. Syst. Man Cybern. C.

[48] Bakar U., Ghayvat H., Hasanm S., Mukhopadhyay S. (2015). Activity and Anomaly Detection in Smart Home: A Survey. Smart Sensors, Measurement and Instrumentation.

[49] Kim E., Helal S., Cook D. (2010). Human Activity Recognition and Pattern Discovery. IEEE Pervasive Comput..

[50] Ghasemi V., Pouyan A.A. Activity recognition in smart homes using absolute temporal information in dynamic graphical models. Proceedings of the 2015 10th Asian Control Conference (ASCC).

[51] Perkowitz M., Philipose M., Fishkin K., Patterson D.J. Mining models of human activities from the web. Proceedings of the 13th Conference on World Wide Web—WWW ’04.

[52] Philipose M., Fishkin K., Perkowitz M., Patterson D., Fox D., Kautz H., Hahnel D. (2004). Inferring Activities from Interactions with Objects. IEEE Pervasive Comput..

[53] Wyatt D., Philipose M., Choudhury T. Unsupervised activity recognition using automatically mined common sense. Proceedings of the Twentieth National Conference on Artificial Intelligence and the Seventeenth Innovative Applications of Artificial Intelligence Conference.

[54] Wong Y.F., Ahmet Sekercioglu Y., Drummond T., Wong V.S. Recent approaches to non-intrusive load monitoring techniques in residential settings. Proceedings of the 2013 IEEE Computational Intelligence Applications in Smart Grid (CIASG).

